# P-2153. Babesia Coinfection is not Associated with Higher Mortality in the United States, 2015-2023: a Retrospective Cohort Study

**DOI:** 10.1093/ofid/ofae631.2307

**Published:** 2025-01-29

**Authors:** Paddy Ssentongo, Natasha Venugopal, Djibril Ba

**Affiliations:** Penn State Hershey Medical Center, Hershey, Pennsylvania; Penn State Hershey Medical Center, Hershey, Pennsylvania; Penn State College of Medicine, Hershey, Pennsylvania

## Abstract

**Background:**

Babesiosis is a tickborne illness caused by the Apicomplexan parasites known as *Babesia spp*, and severe disease is associated with a high mortality rate. Babesiosis is often co-transmitted by other deer-associated zoonoses, including Lyme disease, Ehrlichiosis, and Anaplasmosis maintained by the same vector tick, Ixodes spp. These zoonoses are endemic in the Northeastern United States. Babesiosis-associated coinfection and mortality outcomes are not fully elucidated. The objective of the present study is determining babesiosis coinfection prevalence rates and estimate mortality outcomes.

**Figure:**
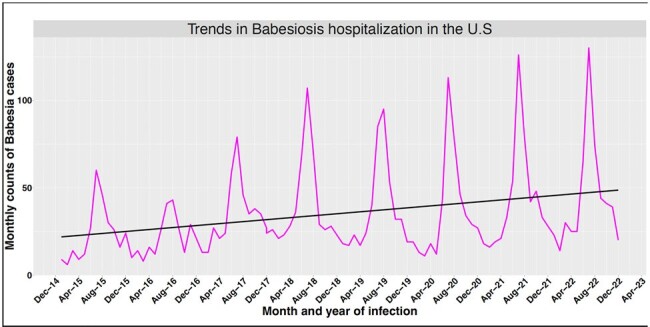

**Methods:**

TriNetX database between 2015 and 2023 was queried for patients with Babesiosis. Cox-proportional hazard model was employed to estimate the mortality risk associated with coinfections (Babesiosis infection co-diagnosed with one or more tickborne illnesses). The analysis focused on Babesiosis coinfection with Lyme disease, Ehrlichiosis, and Anaplasmosis. The exposure was coinfection, and the control group was the Babesia-only population. The primary outcome was a 90-day mortality from the diagnosis of Babesia.

**Figure:**
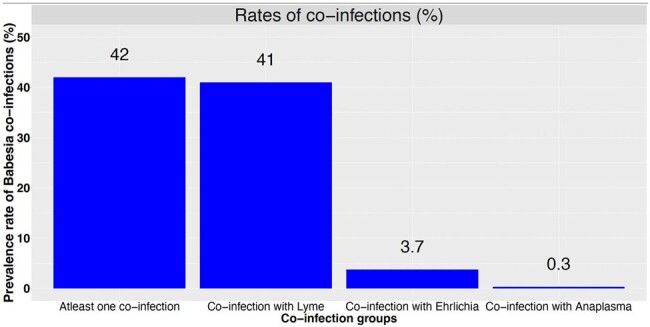

**Results:**

Of the 3521 Babesiosis patients, the mean age was 55 years (SD:18), 51% were male, 75% were White, 2% had asplenia, and 11% had malignancies. Babesiosis cases are on the rise with highest incidence in summer (Fig 1). The prevalence rate of 1 or more co-infection was 42%; 95% CI (40%-43%, Fig 2). Coinfection with Borrelia burgdorferi was the highest with 41%; 95% CI (39%-42%), followed by Ehrlichiosis at 3.7%; 95% CI (3.1%-4.4%). Overall, 90-day mortality was 1.4%; 95% CI (1.0%-1.8%). After adjusting for potential confounders, compared to the coinfections group, the mortality risk was lower in the Babesiosis-only group (HR: 0.43; (95% CI: 0.20-0.90, Fig 3).

**Figure:**
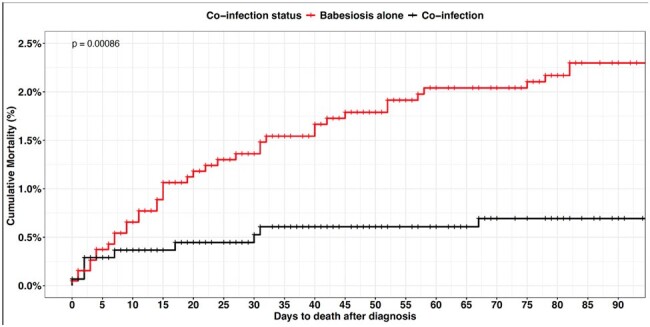

**Conclusion:**

In the extensive study of over 3000 patients with Babesiosis in the United States, the prevalence rates of coinfection were highest with Borrelia burgdorferi, followed by Ehrlichiosis. Rates for Anaplasmosis were low. Coinfection with other tick-borne infections did not increase mortality. It is plausible the findings are due to the likelihood of earlier detection and treatment of coinfection (particularly Lyme disease) with doxycycline.

**Disclosures:**

All Authors: No reported disclosures

